# Expression of *FBN1* during adipogenesis: Relevance to the lipodystrophy phenotype in Marfan syndrome and related conditions

**DOI:** 10.1016/j.ymgme.2016.06.009

**Published:** 2016

**Authors:** Margaret R. Davis, Erik Arner, Cairnan R.E. Duffy, Paul A. De Sousa, Ingrid Dahlman, Peter Arner, Kim M. Summers

**Affiliations:** a The Roslin Institute and Royal (Dick) School of Veterinary Studies, University of Edinburgh, Easter Bush, EH25 9RG, UK; b RIKEN Center for Life Science Technologies (Division of Genomic Technologies) (CLST (DGT)), 1-7-22 Suehiro-cho, Tsurumi-ku, Yokohama, Kanagawa 230-0045, Japan; c Centre for Clinical Brain Sciences, University of Edinburgh, Chancellors Building, 49 Little France Crescent, Edinburgh, EH16 4SB, UK; d Department of Medicine, Huddinge (Med H), Karolinska Universitetssjukhuset Huddinge, 141 86, Stockholm, Sweden

**Keywords:** ADSMC, adipose derived mesenchymal stem cells, BMI, body mass index, CAGE, cap analysis of gene expression, ECM, extracellular matrix, GO, gene ontology, MFS, Marfan syndrome, Adipogenesis, Fibrillin-1, Lipodystrophy, Marfan syndrome, Transcriptomic analysis

## Abstract

Fibrillin-1 is a large glycoprotein encoded by the *FBN1* gene in humans. It provides strength and elasticity to connective tissues and is involved in regulating the bioavailability of the growth factor TGFβ. Mutations in *FBN1* may be associated with depleted or abnormal adipose tissue, seen in some patients with Marfan syndrome and lipodystrophies. As this lack of adipose tissue does not result in high morbidity or mortality, it is generally under-appreciated, but is a cause of psychosocial problems particularly to young patients. We examined the role of fibrillin-1 in adipogenesis. In inbred mouse strains we found significant variation in the level of expression in the *Fbn1* gene that correlated with variation in several measures of body fat, suggesting that mouse fibrillin-1 is associated with the level of fat tissue. Furthermore, we found that *FBN1* mRNA was up-regulated in the adipose tissue of obese women compared to non-obese, and associated with an increase in adipocyte size. We used human mesenchymal stem cells differentiated in culture to adipocytes to show that fibrillin-1 declines after the initiation of differentiation. Gene expression results from a similar experiment (available through the FANTOM5 project) revealed that the decline in fibrillin-1 protein was paralleled by a decline in *FBN1* mRNA. Examination of the *FBN1* gene showed that the region commonly affected in *FBN1*-associated lipodystrophy is highly conserved both across the three human fibrillin genes and across genes encoding fibrillin-1 in vertebrates. These results suggest that fibrillin-1 is involved as the undifferentiated mesenchymal stem cells transition to adipogenesis but then declines as the developing adipocytes take on their final phenotype. Since the C-terminal peptide of fibrillin-1 is a glucogenic hormone, individuals with low fibrillin-1 (for example with *FBN1* mutations associated with lipodystrophy) may fail to differentiate adipocytes and/or to accumulate adipocyte lipids, although this still needs to be shown experimentally.

## Highlights

• *FBN1* mRNA levels are associated with body weight in both humans and mice.
• Fibrillin-1 protein and RNA are produced early in adipogenesis and decline by day 3.
• *FBN1* is co-expressed with mesenchymal markers and proteases during adipogenesis.
• The lipodystrophy region of *FBN1* is highly conserved across mammals.
• The results explain the deficiency of adipose tissue in Marfan syndrome patients.

## 1 Introduction

Fibrillin-1 is a large glycoprotein encoded in humans by the *FBN1* gene (MIM 134797). It is strongly expressed in tissues of mesenchymal origin and localises to the extracellular matrix (ECM) [1], [2] where it contributes to strength and elasticity of tissues [3] and regulates the bioavailability of transforming growth factor beta (TGF-β) [4], [5], [6], [7]. Mutations in *FBN1* result in multisystem abnormalities of connective tissues, most frequently manifesting as Marfan syndrome (MFS) in humans (MIM 154700). MFS affects the skeletal, ocular and cardiovascular systems, with major morbidity and mortality arising from dilatation and dissection of the ascending aorta. In some individuals with *FBN1* mutations, there is also a marked lack of subcutaneous adipose tissue, resulting in an abnormally thin phenotype (for example, see [8]). Extreme cases of lipodystrophy with or without Marfanoid features have been associated with mutations at the 3′ end of the *FBN1* gene [9], [10], [11], [12], [13], [14]. Although this phenotype is not specifically associated with mortality in MFS patients, it causes considerable psychosocial stress, particularly to vulnerable youths who are already struggling to adjust to the diagnosis of a life-threatening condition and have significant body image issues [15], [16]. This body morphology impacts on self-image and on the way patients interact with their peers. At the other end of the scale, obesity affects nearly 10% of the world's population [17] and is a major financial and psychological burden to high income countries. Understanding the normal role of fibrillin-1 in generating adipose tissue could lead to therapies to ameliorate problems relating to both overweight and underweight.

The function of fibrillin-1 in determining the level of adipose tissue has not been rigorously addressed, although a recent paper describes a glucogenic hormone produced from the 170 C-terminal amino acids [13]. The *Fbn1* gene is strongly expressed by mouse cells of adipocyte lineage [1]. Fibrillin-1 is secreted by rat adipocytes [18] and, as mentioned above, the phenotype associated with human *FBN1* mutations frequently (but not always) involves depletion of subcutaneous adipose tissue. A genotype-phenotype correlation exists between the lipodystrophy phenotype and frameshift mutations at the 3′ end of the *FBN1* gene, in the second last exon, coding Exon 64 (exon numbering from http://www.umd.be/FBN1/; shown as Exon 65 in the Ensembl Genome Browser) [9], [10], [11], [12], [13], [14]. Reduced subcutaneous tissue and abnormal adipocytes can also be associated with mutations in central exons, for example in our patient with a mutation in exon 25 [8], clearly indicating that fibrillin-1 is involved in determining the formation and maturation of adipocytes.

Adipose tissue develops through a cascade of events leading to conversion of mesenchymal stem cells to preadipocytes which undergo terminal differentiation into adipocytes. This process is regulated by key transcription factors CEBP (CCAAT enhancer binding protein) and PPARG (peroxisome proliferator activated receptor gamma) [19], [20]. Once proliferation of adipocyte precursors has ceased, lipid-filled storage vacuoles form in the intracellular space [21]. Adipocytes are constantly replenished in adult tissues (approximately 10% per annum [22]) and response to alterations in nutritional status can involve changes in both cell size and number (for example, [23], [24], [25]). Understanding the factors regulating adipocyte differentiation offers the potential of treatments for obesity (adipose excess) as well as lipodystrophy (adipose deficiency).

Adipogenesis can be seen as having two phases (reviewed extensively by [26]). In the early phase cells become committed to differentiation and in the later phase cells expand to accommodate the requirements of lipid storage. The ECM is extensively reorganised during this process, with down-regulation of most secreted proteins and up-regulation of basement membrane and basal lamina. Over 65 proteins make up the ECM of adipose tissue [26] including fibrillin-1, fibronectin, a range of collagen subunits, osteonectin and latent transforming growth factor binding protein 1 (LTBP1), a member of the fibrillin gene superfamily. During preadipocyte formation from mesenchymal stem cells collagen type VI increases in amount and provides a scaffold for a lipid monolayer. The extracellular matrix of the preadipocytes then undergoes gradual up-regulation of collagen type IV [27], which interacts with collagen type VI, and collagen type V. Fibrillar collagens (type I and type III), fibronectin and other ECM components may peak early in differentiation before being down-regulated. As differentiation progresses, the ECM is reorganised to provide storage space for lipid vacuoles. The ECM in mature adipose tissue is under constant turnover to ensure that adequate lipid storage space is available [26], [28].

The process of adipogenesis can be recreated *in vitro* using primary mesenchymal stem cells, treated with growth factors to promote differentiation along the adipose lineage. In this paper we describe investigations of the role of fibrillin-1 in adipogenesis *in vitro* and adipose expansion *in vivo*, based on independent experiments using either microarray or promoter expression analysis derived from the FANTOM5 project [29], [30].

## 2 Materials and methods

### 2.1 Analysis of mouse gene expression across strains

Mouse gene expression data were downloaded from BioGPS [31]. Gene expression in mouse epididymal adipose tissue was derived from data presented in [32], based on a customised microarray platform GNF1M. There was one probeset for *Fbn1* (gnf1m00711_a_at) and one for *Fbn2* (gnf1m02242_a_at). Available results were from pooled RNA obtained from mice at 25 weeks of age, of the following mouse strains: C57BL/6J (*n* = 3), C3H/HeJ (*n* = 4), CBA/J (*n* = 2), DBA/2J (*n* = 3). To find genes with similar expression patterns to *Fbn1*, the Correlation function in BioGPS was used, with minimum correlation coefficient set at 0.75. Mouse phenotype data were obtained from the Mouse Phenome Database (MPD; [33]) which provides results for mice scanned immediately post-mortem using mouse densitometer dual energy X-ray absorptiometry (PIXImus mouse densitometer (LUNAR, Madison, WI). Details of the mouse husbandry are available at http://phenome.jax.org/db/q?rtn=projects/docstatic&doc=Jaxpheno1/Jaxpheno1_Animal. Results for MPD data set Jaxpheno1 were downloaded for body weight, body fat tissue weight and body fat percentage for males and females aged 8 and 16 weeks. Results for single nucleotide polymorphisms for C57Bl/6J, C3H/HeJ, CBA/J and DBA/2J strains were retrieved from MPD and the Mouse Genome Informatics database (MGI; [34]). The mouse *Fbn1* transcription start site region was identified using data from the FANTOM3 [35] and FANTOM5 projects and the coordinates on the current build of the mouse genome (GRC m38.p4) determined by a BLASTN search in Ensembl [36].

### 2.2 Analysis of human adipose tissue gene expression data

Gene expression data from the adipose tissue of a previously analysed cohort of 30 obese (BMI > 30 kg/m^2^) and 26 non-obese (BMI < 30 kg/m^2^) women [37] was subjected to Significance Analysis of Microarrays [38]. Affymetrix microarray data from 114 adult Swedish women without diabetes [39] was examined for correlations between markers of adiposity and *FBN1* expression with the statistical package Statview (SAS Institute Inc., NC, USA). The human *FBN1* transcription start site was identified using data from the FANTOM3 and FANTOM5 projects [2], [29], [35].

### 2.3 Detection of fibrillin-1 protein during adipogenesis

To assess further the impact of adipogenesis on fibrillin1, cryopreserved adipose derived mesenchymal stem cells (ADSMC) from a single female donor who had undergone abdominal liposuction were obtained from the Edinburgh Adipose Tissue Bank, University of Edinburgh, UK [40]. The research was approved by the South East Scotland Research Ethics Committee 03 (Reference 1-0/S1103/45). The cells were cultured initially in DMEM (Gibco, Life Technologies Lit, Paisley UK) with 10% heat inactivated foetal bovine serum (GE Healthcare, Little Chalfont, UK), 1 X GlutaMAX (Gibco), 1 μg/μL bFGF (PeproTech, Rocky Hills, NJ, USA), at 37 °C and 5% CO_2_. Culture vessels were coated with 0.1% gelatin. Seeding density was 20,000 cells per well when using 8-well chamber slides (NUNC) and 50,000 cells per well when using 6 well plates. StemPro® Adipogenesis Differentiation Medium (Fisher Scientific, Loughborough UK) was prepared according to manufacturer's instructions and filtered through a 500 mL 75 mm 0.45 μm filter unit (Thermo Scientific, Leicestershire, UK). Cells were transferred to this medium after 24 h when 75–80% confluency had been achieved. 100 μL (chamber slides) or 1 mL (6 well plates) of StemPro® Adipogenesis Differentiation Medium was then added to the cells while DMEM with foetal bovine serum, GlutaMAX and bFGF as above was added to the control (non-differentiating) samples. Samples for immunocytochemistry (in the 8-well chamber slides) were fixed with 95% ethanol, 5% acetic acid for 20 min at − 20 °C. Slides were stained for fibrillin-1 as described previously [2] using a mouse monoclonal anti-fibrillin-1 antibody (11C1.3 ab3090, Abcam, Cambridge, UK) and AlexaFluor-488 labelled goat anti-mouse anti-IgG second antibody (Invitrogen, Paisley, UK).

Oil Red O stock solution was prepared with 0.7 g Oil Red O (Sigma-Aldrich) and 200 mL isopropanol, stirred overnight and filtered through a 0.2 μm filter unit. The solution was stored at room temperature and a working solution was prepared at 6:4 (v/v) Oil Red O stock solution and distilled water. This was incubated at room temperature for 20 min and then filtered through a 0.2 μm Millex syringe filter unit (Millipore, Cork, Ireland). Samples for Oil Red O staining (in 6-well plates) were fixed with 10% neutral buffered formalin solution (Sigma Aldrich) for 5 min, and then in fresh formalin solution at room temperature for 1 h. The wells were briefly washed with 60% isopropanol and allowed to air dry at room temperature. 200 μL of Oil Red O working solution was added to each well, for 10 min at room temperature, then the wells were washed with distilled water four times for 10 min. Wells were imaged using an Axio Lab.A1 microscope (Zeiss).

### 2.4 CAGE analysis of *FBN1* expression during human adipogenesis

We examined gene expression and promoter usage in humans from a publicly available time course of adipogenesis using a different donor. Details of this experiment have been published elsewhere (Auxiliary File S1 of [30]) and the data are available for download at the FANTOM5 website [30]. Briefly, adipose-derived stem cells were extracted from the stromal vascular fraction of subcutaneous white adipose tissue from a single male donor, expanded and subcultured *in vitro* and treated to undergo differentiation to adipocytes [41], [42], [43]. RNA was harvested for cap analysis of gene expression (CAGE) analysis as part of the FANTOM5 project [29], [30]. Following the FANTOM5 quality control process [29], [30], some time points were removed for some replicates. For the present analysis, the time points analysed were Replicate 1: 3 h, 1 day, 2 days, 4 days, 8 days, 12 days, 14 days post induction of differentiation; Replicate 2: 0 h, 2 days, 8 days, 12 days, 14 days post induction; Replicate 3: 0 h, 3 h, 1 day, 2 days, 4 days, 8 days, 12 days post induction. In addition, another set of samples from the same study was examined for *FBN1* expression during adipogenesis. These samples consisted of adipocyte precursor cells isolated from the adipose stromal vascular fraction of four additional donors, differentiated without cell passage and sampled at three time points (day 4, day 8 and day 12). The gene based and promoter based data for these experiments were downloaded from the FANTOM5 website. Gene expression patterns were clustered using BioLayout *Express*^3D^ [44], [45]. Pearson correlation coefficients and MCL inflation values used for different analyses are given in the results. Gene Ontology (GO) term enrichment in the generated clusters was analysed using the Database for Annotation, Visualisation and Integrated Discovery (DAVID) v6.7 [46] using total human genes as background.

### 2.5 Evolutionary conservation of the lipodystrophy region of *FBN1*

Predicted amino acid sequences of exon 64 (based on the human sequence) of fibrillin-1, fibrillin-2 and fibrillin-3 were extracted from the Ensembl database for the following species: human (*Homo sapiens*), mouse (*Mus musculus*), sheep (*Ovis aries*), ferret (*Mustela putorius furo*), bushbaby (*Otolemur garnettii*), armadillo (*Dasypus novemcinctus*), panda (*Ailuropoda melanoleuca*), chicken (*Gallus gallus*), flycatcher (*Ficedula albicollis*), turkey (*Meleagris gallopavo*), coelacanth (*Latimeria chalumnae*), fugu (*Takifugu rubripes*), medaka (*Oryzias latipes*), spotted gar (*Lepisosteus oculatus*), stickleback (*Gasterosteus aculeatus*), tetraodon (*Tetraodon nigroviridis*), tilapia (*Oreochromis niloticus*) and Chinese soft shell turtle (*Pelodiscus sinensis*). The sequences were aligned using ClustalW (DNASTAR, Lasergene, Madison WI USA). Percent identity was based on sequence displacement values determined by the ClustalW alignment. Conserved amino acid residues were identified manually within the alignments.

## 3 Results

### 3.1 *Fbn1* gene expression correlates with amount of adipose tissue in mouse

**Fig. 1:**
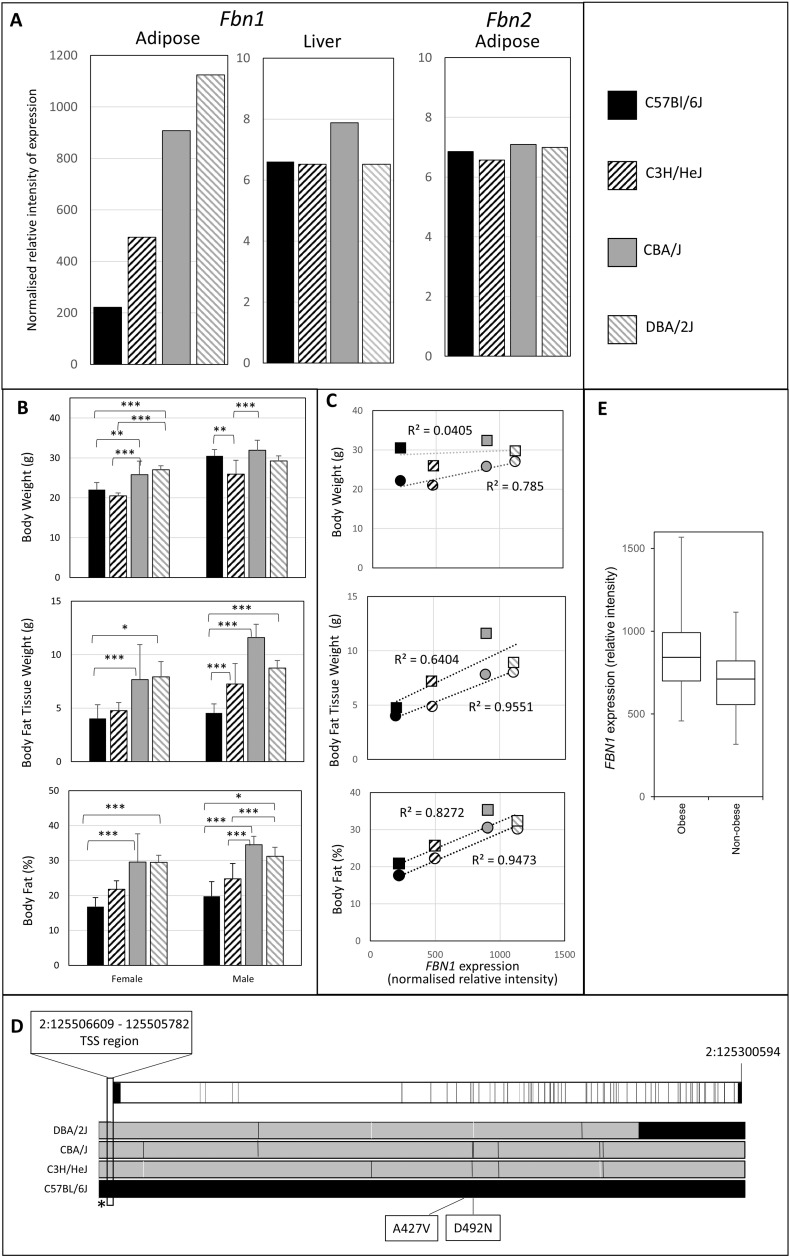
Fibrillin gene expression in adipose tissues. A. *Fbn1* (probeset gnf1m00711_a_at) and *Fbn2* (probeset gnf1m02242_a_at) expression levels in tissues of four strains of mice. Data derived from [32] and available from BioGPS. Black — C57Bl/6J; hatched black — C3H/HeJ; grey — CBA/J; hatched grey — DBA/2J. B. Body weight, body fat tissue mass (head excluded) and body fat percent for four strains of mice. Data derived from MPD, dataset jaxpheno1). Symbols are coded as for panel A. Bars show significance (ANOVA). ***: *p* < 0.001; **: 0.001 < *p* < 0.01; *: 0.01 < *p* < 0.05 C. Correlation between body weight, body fat tissue mass (head excluded) and body fat percent and *Fbn1* expression. Circles represent females and squares males. Trendlines and regression coefficients were calculated using Microsoft Excel 2013. Symbols are coded as for panel A. D. Single nucleotide polymorphism alleles in the mouse *Fbn1* gene. Upper bar shows the structure of the gene with exons shown in black and introns in white. Lower bar shows the genotype of C3H/HeJ, CBA/J and DBA/2J mouse strains compared with C57BL/6J shown in black. White bars indicate missing data. Asterisk shows the location of a single base insertion relative to C57BL/6J.Two non-synonymous coding variants are shown. C57BL/6J mice have D429 while DBA/2J mice have N429. C47BL/6J mice have A427 while the other lines have V427. E. *FBN1* expression in obese (left) and non-obese (right) human females. Boxes show the boundaries of the first and third quartiles; bar shows the median value and whiskers show the minimum and maximum values. An average difference of 21% at a false discovery rate of 0.05 was found.

The expression data available at BioGPS for different mouse strains indicate considerable variation in expression of *Fbn1* in epididymal adipose tissue from males of different mouse strains (Fig. 1A). The expression of the fibrillin gene family member *Fbn2* was very low in adipose tissue and *Fbn1* expression was much lower in other tissues such as liver (Fig. 1A), hippocampus and pancreas across strains. To assess the impact of the variability of *Fbn1* expression in adipose tissue, we used data available through MGI. We compared results for four strains of mice: DBA/2J (high expression of *Fbn1*), CBA/J (moderate to high expression), C3H/HeJ (low to moderate expression) and C57Bl/6J (low expression) (Fig. 1A). At 16 weeks, female C57BL/6J and C3H/HeJ were not different in body weight and nor were female CBA/J and DBN/2J. However female C57BL/6J and C3H/HeJ were both significantly lower in body weight than CBA/J and DBA/2J (Fig. 1B). Male C3H/HeJ were lower in body weight than C57BL/6J and C3H/HeJ were also lower than CBA/J male mice. This suggested a possible relationship between body weight and *Fbn1* levels, particularly for females. To determine whether this was due to differences in adipose tissue, we examined body fat tissue weight and body fat percent in both males and females at 16 weeks (Fig. 1B), with C57Bl/6J showing a significant difference from CBA/J and DBA/6J for both males and females. C3H/HeJ tended to have intermediate values. We then investigated correlations between measures of body fat and *Fbn1* expression level, using the data available at BioGPS. *Fbn1* probeset gnf1m00711_a_at (measured in epididymal fat) showed a high correlation with measures of body fat for both males and females at 16-weeks of age (Fig. 1C). These results suggest that there is an association between *Fbn1* mRNA levels and the deposition of adipose tissue in mouse.

Analysis of SNP alleles in the four mouse strains (see Section 2.1) showed that C57BL/6J carried a different haplotype for the majority of the *Fbn1* gene sequence (Fig. 1D), although DBA/2J shared this haplotype at the 3′ end of the gene. There were two missense mutations resulting in a coding change where the genotype differs between C57BL/6J and DBA/2J, one where CBA/J and C3H/HeJ also carry the C57BL/6J allele. No splice junction or stop codon mutations were segregating in these four strains, and the majority of the segregating SNPs were intronic. C57Bl/6J mice also had a single base deletion just upstream of the transcription start site region. No other promoter region differences were found. The extensive genetic difference between the *Fbn1* genes of the C57BL/6J strain and the other three strains may explain the lower *Fbn1* expression level in this strain. There were minimal differences among the other three strains except at the 3′ end of the gene (Fig. 1D).

In order to find genes that were coexpressed with *Fbn1* in mouse adipose tissue, we identified those whose expression pattern was correlated with *Fbn1* (correlation coefficient ≥ 0.75) across all mouse strains available for the eQTL analysis using the Correlation function of BioGPS. The list (available in [47]) included a number of genes previously found to be coexpressed with *Fbn1* in C57BL/6 mice across a wide range of cell types [1] (*Bgn*, *Cd248*, *Col1a2*, *Col3a1*, *Col4a1*, *Col4a2*, *Col5a1*, *Fn1*, *Fstl1*, *Lox*, *Ppic*, *SerpinH1*, *Sparc*), indicating that these genes may be under similar regulatory control.

### 3.2 *FBN1* gene expression correlates with markers of adiposity in humans

*FBN1* mRNA levels were significantly up-regulated on average in abdominal white adipose tissue of obese women subjects compared with non-obese women (21% at false discovery rate 0.05) [37] (Fig. 1E). As with the mice, *FBN2* was not differentially expressed in obese compared with normal women in this study (not shown). In a separate cohort of Swedish women [39], there was a small but significant correlation between *FBN1* expression levels (measured by expression microarray) and body mass index, percentage body fat and fat cell volume (Table 1). Body mass index, percentage body fat and fat cell volume were highly correlated with each other and the correlations with *FBN1* mRNA are not independent. *FBN1* level was not correlated with fat cell number (Table 1). This suggests that fibrillin-1 is involved in determining human fat mass and that a high level is associated with expansion of hypertrophic adipose tissue through increased fat cell volume rather than number. > 15.000 genetic variants have been detected in the human *FBN1* gene (see, for example, dbSNP; [48]), many in the promoter region, which could be associated with the differential expression in human subjects.

### 3.3 Fibrillin-1 protein disappears as human ADMSC undergo differentiation

**Fig. 2:**
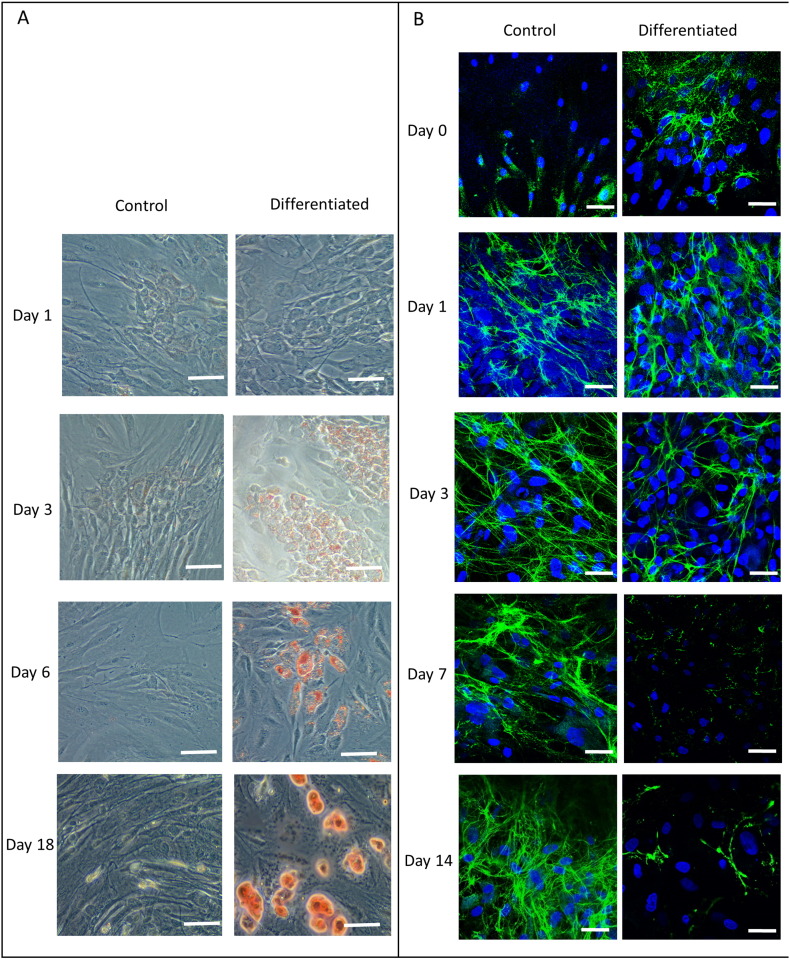
Human *in vitro* adipogenesis time course. A. Oil Red O staining showing formation of lipid droplets. Adipose derived mesenchymal stem cells (ADMSC), untreated (left panel) or differentiating to adipocytes (right panel) stained with Oil Red O at 1 d, 3 d, 6 d, 8 days and 18 days after induction of differentiation. Red staining shows lipid droplets (darker grey surrounded by bright ring in print version). Images show results representative of three separate experiments. 0 time was similar to 1 day time point. Scale bars represent 50 μm. B. Fibrillin-1 immunocytochemistry. ADMSC untreated (left panel) or undergoing differentiation (right panel) were stained using a fluorescently-labelled anti-fibrillin-1 antibody. Nuclei are stained blue with DAPI and fibrillin-1 is shown by green fluorescence (white in print version). Images show results representative of three separate experiments. Scale bars represent 20 μm. (For interpretation of the references to color in this figure legend, the reader is referred to the web version of this article.)

Since the examination of *Fbn1* gene expression in mouse and human suggested a correlation with the level of adipose tissue, we next examined *FBN1* gene expression during a time course of human adipogenesis *in vitro*. We used a sample of adipose derived mesenchymal stem cells (ADMSC) which were stimulated to undergo differentiation in the adipocyte lineage, and compared fibrillin expression level with that in an undifferentiated culture of the same cells. Three replicates from a single donor were studied. The differentiated ADSMC cultures showed increasing amounts of Oil Red O staining (indicative of lipid production) as the time course progressed (Fig. 2A), with lipid droplets appearing in unstained cultures by day 3, accumulating in number and size up to day 18. Small oil droplets were also visible within the cytoplasm of unstained cells (not shown) by day 18. Non-differentiated controls showed minimal red staining at the later time points (day 6 and day 18) (Fig. 2A). Therefore, the adipogenic differentiation appeared to have been successful.

Fluorescent immunocytochemistry was performed for fibrillin-1 protein during the adipogenesis time course. This revealed that both treated and control samples had a developing network of fibrillin-1 microfibrils at day 1 (that is, after 24 h in normal medium and a further 24 h in adipogenesis medium). For the untreated cells, the matrix increased over the time course and by day 14 there was an extensive presence of fibrillin-1 microfibrils as seen in Fig. 2B.This matrix was similar to that previously described for fibroblasts [2], osteosarcoma cells [49] and chondrocytes (MR Davis, unpublished results). In contrast, the differentiated cells showed initial formation of fibrillin-1 microfibrils at day 1, but these did not elaborate and by day 3 had begun to disappear. At day 7 and day 14 there was very little evidence of fibrillin-1 in the extracellular matrix of the treated cells (Fig. 2B).

### 3.4 *FBN1* mRNA is down-regulated early in adipogenesis

**Fig. 3:**
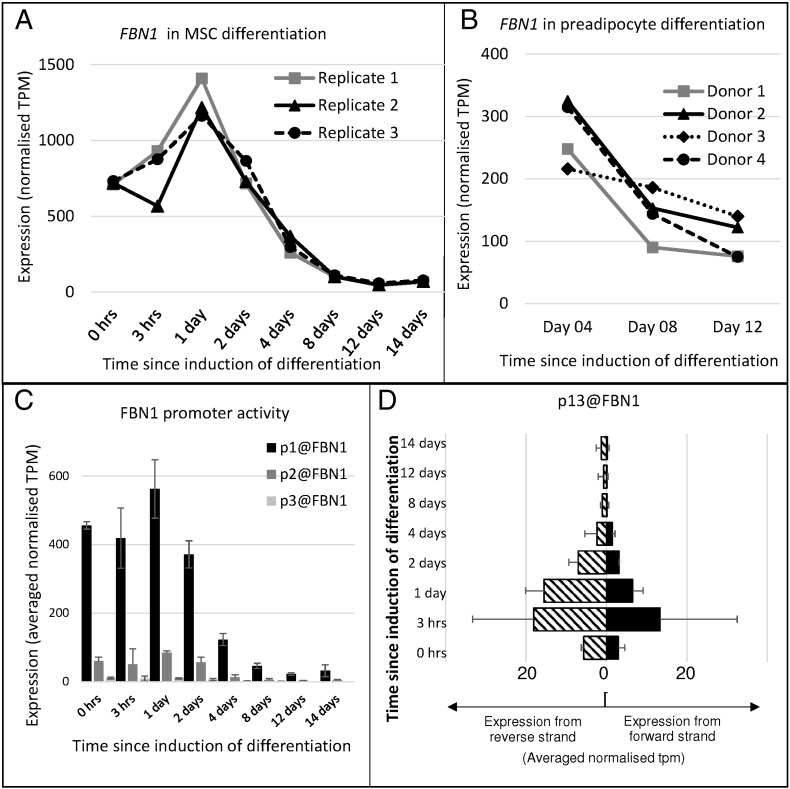
Fibrillin expression and promoter usage during adipogenesis. Expression levels were derived from transcription start site data [30] available at FANTOM5 website. A. *FBN1* mRNA levels during the *in vitro* time course of adipogenesis from mesenchymal stem cells. Y axis shows the gene-based expression level, given as normalised tags per million (tpm); X axis shows the time points. Each replicate is shown separately. B. *FBN1* levels during preadipocyte differentiation to adipocytes for four donors. Y axis shows the gene-based expression level, given as normalised tpm for each donor; X axis shows the time points. C. *FBN1* promoter usage during adipogenesis. Promoters are numbered according to expression level across the whole FANTOM5 data set [29], [30], so p1@FBN1 is the highest expressing *FBN1* promoter over the whole data set. Y axis shows the promoter-based expression level, given as normalised tags per million (tpm) averaged over three replicates; X axis shows the time points. Error bars show 1 standard deviation. D. Expression of p13@FBN1, showing bidirectional expression pattern (averaged normalised tpm). *FBN1* is encoded on the reverse strand (left side of graph). Error bars show 1 standard deviation.

The analysis of fibrillin-1 protein suggested that the amount of fibrillin declines rapidly after the onset of adipogenic differentiation (Fig. 2B). Preliminary analysis using gene expression microarary and quantitative reverse transcriptase polymerase chain reaction showed that there was a decline in *FBN1* mRNA during the differentiation time course used for immunofluorescence staining (not shown). To examine further the pattern of *FBN1* mRNA expression during adipogenesis, we analysed a gene expression dataset which explored a similar but more extensive time course of adipogenic differentiation from mesenchymal stem cells. This study, which has been described previously [30], looked at gene expression using Cap Analysis of Gene Expression (CAGE), a quantitative method to assess promoter usage and hence gene expression [50]. The three replicates (from a single donor) of this study were used to examine the expression of fibrillin genes during adipogenesis from mesenchymal stem cells. Validation that adipogenesis had occurred is described elsewhere (Auxiliary Table S2 of [30]). Gene expression results (given as normalised tags per million, tpm) from RNA sampled at time points between 3 h and 14 days after initiation of differentiation were used. *FBN1* mRNA level increased slightly in the first two days of differentiation but then declined considerably by day 4 (Fig. 3A). Analysis of *FBN1* expression in preadipocytes (from four different donors) differentiated into adipocytes and sampled at three time points (day 04, day 08 and day 12) further confirmed consistent and significant down-regulation of *FBN1* during human adipogenesis (Fig. 3B). Thus cells from six different donors showed consistent down-regulation of fibrillin-1 mRNA or protein after several days in differentiation medium.

Since we had previously shown some differential promoter usage by fibrillin genes in different cell types [2], we examined *FBN1* promoter usage in the MSC-adipocyte differentiation time course. There was no evidence of promoter switching during the time course (Fig. 3C). The highest expressing *FBN1* promoters (p1@FBN1, p2@FBN1 and p3@FBN1) were down-regulated in all replicates as differentiation proceeded. The remaining promoters showed very low activity, except for p13@FBN1 which exhibited roughly balanced bilateral expression in all three replicates in the first 24 h (Fig. 3D), suggesting that it may have enhancer activity in this system [51]. In the sample of adipose nuclei in the Roadmap Epigenomics Project [52], there was evidence of monomethylation of Histone 3 lysine 4 (H3K4me1; frequently found at enhancers) and acetylation of Histone 3 lysine 27 (H3K27ac; found at promoters and enhancers) [53] at a higher level in the region of p13@FBN1 than p1@FBN1. Leucocytes had very low levels of these epigenetic marks, consistent with the tissue specificity of enhancer histone modification patterns [54].

### 3.5 *FBN1* is coexpressed with other mesenchymal genes during human adipogenesis *in vitro*

Using the full gene expression data set from the previous study [30] we generated a network graph containing 7252 nodes (genes) connected by 688,617 edges (correlation in expression pattern of 0.85 or greater) with BioLayout *Express*^3D^. Clustering with an MCL inflation value of 2.2 generated 29 coexpression clusters of 10 or more nodes. The list of genes in each cluster is available in [47]. The network layout and average expression profile of up- and down-regulated clusters are shown in [47]. There was a temporal transition from Cluster 02 (genes that are down-regulated rapidly early in differentiation) to Cluster 04 (genes down-regulated more gradually including *FBN2* and *FN1* encoding fibronectin), Cluster 06 (genes whose expression persists through the first two days of differentiation, including *FBN1*) and Cluster 03 (genes with a peak of expression after the initiation of differentiation). Cluster 07 and Cluster 01 contain genes that are expressed in differentiated adipocytes, and peak at the end of the differentiation time course. The *FBN1* gene was found in a cluster of genes that were down-regulated after 1–2 days in culture. In addition to *FBN1*, Cluster 06 contained genes associated with GO terms for ECM, cell adhesion, mesoderm development, endoplasmic reticulum and osteoblast [47].

We have previously shown coexpression of connective tissue genes with *FBN1* in mouse and humans [1], [2]. A number of these were down-regulated during adipogenesis. Clustered tightly with *FBN1* in this analysis was a set of mesenchymal genes previously been shown to be coexpressed with mouse and human *FBN1*,including *BGN*, *COL1A1*, *COL1A2*, *COL6A1*, *COL6A2*, *COL16A1*, *EFEMP2*, *FKBP10*, *LIX1L*, *MRGPRF*, *NFATC4*, *TIMP1* and *TIMP2* [1], [55], [56]. The down-regulated cluster which contained the related gene *FBN2*, also contained other genes often coexpressed with *FBN1* (for example, *ANTXR1*, *CALU*, *COPZ2*, *FN1*, *LOX*, *LTBP2*, *PTGIS*, *TIMP3*, *VGLL3*) [1]. The largest down-regulated cluster contained *ACTA2*, *ATOH8*, *CALD1*, *LOXL1*, *PALLD*, *POSTN*, *PRRX1*, *PRRX2*, *PRSS23*, *PTX3*, *TGFB3*, *TGFB1I1* and *THBS1*, which have also been shown to be coexpressed with mouse *Fbn1* [1]. The coexpression of genes commonly associated with *FBN1* indicates that general mesenchymal genes were shut down during adipogenesis, while mesenchymal genes encoding proteins specific to the specialised needs of adipose connective tissue, such as those encoding collagen type IV subunits, were up-regulated, as shown previously [30].

We analysed the dataset to identify transcription factors that might be involved in regulating the level of *FBN1* during adipogenesis. We created a network layout for transcription factor gene expression using BioLayout *Express*^3D^ and identified a cluster of transcription factors that were down-regulated during adipogenesis (data available in [47]). DAVID analysis showed that these included transcription factors associated with embryonic development, and development of specialised adult tissues including bone, lung, nervous system and haematopoietic cells. Genes for transcription factors that were down-regulated just ahead of or with the down-regulation of *FBN1* (from the initiation of adipogenesis, or within the first 24 h) included a number that have previously been associated with regulation of *FBN1*, either because of the presence of a binding motif in the promoter region [35] (for example *TEAD1*, *2* and *4*, *RUNX1* and *2*, *KLF4*, *SNAI2*, *CEBPD* and many genes encoding forkhead box (FOX) transcription factors) or because of motif activity [1] (*CIZ1*, *PRDM1*, *EGR1*, *2* and *3*, *ATF6B*, *E2F4* and *NFATC1* and *2*).

We also looked at genes coding for proteases that are involved in processing and degrading fibrillins. Profibrillin molecules are cleaved to the active form by furin [57], [58], [59]. The *FURIN* gene that encodes this protease showed very low expression throughout the time course. In contrast, genes encoding proteases that degrade fibrillins, including *MMP2*, *MMP3* and *MMP14* [60], were highly expressed and peaked in activity at day 2, consistent with the disappearance of fibrillin-1 microfibrils by day 3 (profile shown in [47]). Other matrix metalloproteinase genes (*MMP1*, *MMP11*) were down-regulated while *MMP15* was up-regulated, suggesting specific roles for these enzymes during adipogenesis. Three genes encoding inhibitors of MMPs (tissue inhibitor of matrix metalloproteinase; TIMP) were found to be down-regulated (*TIMP1* and *TIMP2* clustering with *FBN1* and *TIMP3* with *FBN2*) while *TIMP4* was up-regulated by day 4 of differentiation. These results indicate that the amount of fibrillin-1 protein is likely to be controlled by expression of *FBN1* mRNA, by the presence of the processing enzyme furin and by the balance between the degradative proteases and their inhibitors. The net effect is to reduce the level of fibrillin as differentiation proceeds.

### 3.6 The lipodystrophy region of *FBN1* has been highly conserved through vertebrate evolution

**Fig. 4:**
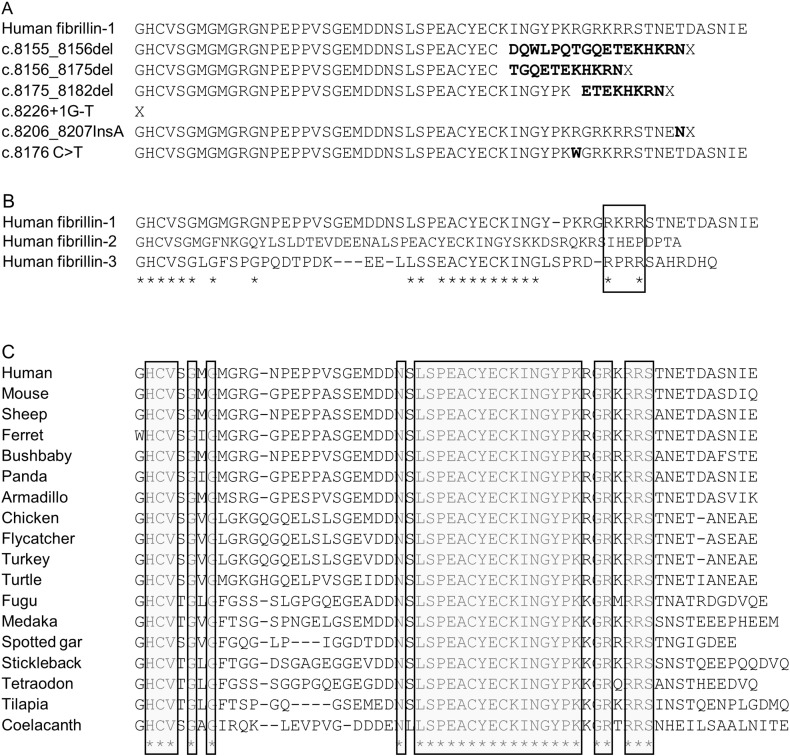
Conservation of the amino acid sequence of coding exon 64 of fibrillin proteins. A. Effect of five unique protein truncation mutations in human *FBN1* on exon 64 sequence. Sequences were obtained from [9], [10], [11], [12], [13], [14]. Mutant sequences are shown in bold and X represents an in frame termination codon. Also shown is a missense mutation c. 8176C > T which results in substitution of arginine at position 2726 with tryptophan (p.R2726W; bolded W) which produced the skeletal phenotype of Marfan syndrome [65]. B. Amino acid sequence of exon 64 of human fibrillin-1, fibrillin-2 and fibrillin-3. Conserved residues are shown by asterisks below the sequences. Furin cleavage site is boxed. Data were derived from the Ensembl data base and sequence similarities determined using ClustalW as described in the methods. C. Amino acid sequence of Exon 64 of fibrillin-1 in a range of vertebrate species. Conserved residues are shown by asterisks below the sequences and are boxed in grey. Data were derived from the Ensembl data base and sequence similarities determined using ClustalW as described in the methods. All sequences were determined to be homologous to human coding exon 64 based on amino acid sequence homology and C-terminal location within the predicted protein.

Seven patients with severe generalised lipodystrophy have been reported to have mutations in coding exon 64 of *FBN1* [9], [10], [11], [12], [13], [14]. All mutations resulted in premature stop codons (Fig. 4A) and were concordant for loss of the C-terminal end of exon 64 and all of exon 65. Unlike the majority of the *FBN1* exons [49], [61], [62] the amino acid sequence encoded by Exon 64 was not highly similar among the three human fibrillin proteins, fibrillin-1, fibrillin-2 and fibrillin-3 (Fig. 4B), except that the furin cleavage site (consensus sequence R-X-K/R-R) [63], [64], was retained. However, the entire exon was strongly conserved in fibrillin-1 across mammalian species with some reduction in similarity in more distantly related vertebrates (Fig. 4C; see also Fig. S1 of [13]). In fibrillin-1, there was striking conservation of the furin cleavage site and of the 15 amino acids immediately upstream of the common deleted region. All lipodystrophy mutations produced frame shifts that would result in termination of the protein before or just after the furin cleavage site, with loss of the glucogenic fragment [13]. An additional patient has been reported with a missense mutation p.R2726W, which occurs immediately before the furin cleavage site (Fig. 4A) and affects an arginine that is conserved across mammals and birds (Fig. 4C). This mutation was shown to prevent proteolytic cleavage of the protein [65]. The affected individual had isolated skeletal manifestations of MFS; there is no information about the level of subcutaneous fat.

## 4 Discussion

In this study we have analysed several publicly available databases and a cell culture model of adipogenesis to examine the possible role of fibrillin-1 in adipogenesis. Firstly we showed that *Fbn1* level in mouse strains is correlated with the amount of adipose tissue (measured as total adipose or as percent fat). This relationship was true although the mRNA was derived from epididymal fat from male mice at 25 weeks and the weight and body fat composition were from both male and female mice at 16 weeks, indicating that it is likely to be a general feature of mouse adipose tissue and that fibrillin-1 is important to the synthesis and/or maintenance of adipose tissue *in vivo*. There was no similar relationship with the fibrillin family member *Fbn2* (not shown), so this association appeared to reflect a specific role of fibrillin-1. Genetic variation in the promoter region of the *Fbn1* gene may explain the differences in expression between DBA/J (high expression) and C57BL/6J (low expression). Higher *FBN1* expression was also found in obese women than non-obese women and there was a significant correlation between *FBN1* expression level and several measures of adiposity (including cell volume but not cell number) in 114 human female subjects. These results are consistent with the finding of reduced subcutaneous tissue with abnormal adipocytes in some Marfan syndrome patients (for example [8]) and in patients with *FBN1* mutations causing lipodystrophy [9], [10], [11], [12], [13], [14].

We then used a cell culture model of adipogenesis to explore the timing of fibrillin-1 expression during adipogenesis. In this model isolated human adipose derived mesenchymal stem cells were triggered to form adipocytes over a period of two weeks. Fibrillin-1 protein was present throughout the period in untreated cells which maintained their undifferentiated mesenchymal stem cell phenotype, but in treated cells the protein was present early in the time course and then disappeared, coinciding with the appearance of oil droplets (confirmed by Oil Red O staining) from day 3. To examine gene expression, we used publicly available data from a comparable time course [30] to confirm the decrease in mRNA level in differentiating mesenchymal stem cells. Similarly, during the transition of preadipocytes from four donors to adipocytes [30] a consistent decrease of *FBN1* mRNA was seen. *FBN1* was one of a number of mesenchymal genes where the expression dropped after 24 h in differentiation medium and the level remained low up to day 14. Many of these down-regulated genes had previously been seen to be coexpressed with *FBN1* [1], [2] and a number encoded transcription factors that have been suggested to regulate *FBN1* [1], [35]. Adipogenesis appears to involve the down-regulation of generic ECM genes in this group and up-regulation of specialised ECM genes (including *ADAMTS18*, *COL4A1*, *COL4A2*) coding for proteins which facilitate the dynamic storage of lipid in adipose tissue. Taken together these results suggest that fibrillin-1 is associated with the establishment of mesenchymal stem cell commitment but not terminal differentiation, at least in the adipocyte lineage. The elevated expression seen in mouse and human tissues, in contrast to decline during the synchronised adipogenesis of the *in vitro* culture model, indicates that there is likely to be a homeostatic role for fibrillin-1 in mature adipose tissue. *FBN1* level was correlated with cell volume rather than cell number, indicating that the role of fibrillin-1 is likely to be in aiding the metabolic and structural changes necessary for lipid deposition rather than in cell proliferation.

Coding Exon 64 of the *FBN1* gene is important for the role of fibrillin-1 in human adipose tissue, since individuals with mutations truncating the protein at this point have severe lipodystrophy. Although this region was not conserved across the three human fibrillins (other than the furin cleavage site), it was highly conserved throughout the mammalian *FBN1* genes (see also Fig. S1 of [13]). This suggests that the role in adipogenesis is specific to fibrillin-1, consistent with the low level of *FBN2*/*Fbn2* mRNA in human and mouse adipogenesis. The peptide beyond the furin cleavage site, which would be absent in the lipodystrophy patients, is also essential for the secretion of fibrillin-1; for some patients proteins lacking the C terminal amino acids were retained within the cell [66].

It has recently it has been shown that the C-terminal 170 amino acids of fibrillin-1 (beyond the furin cleavage site) form a peptide hormone, now called asprosin [13], which shows a higher vertebrate similarity score than other fibrillin-1 exons. Asprosin is released from white adipose tissue in response to low dietary glucose and stimulates the liver to release glucose, triggering insulin release from the pancreas. This discovery is consistent with the correlation between *FBN1* expression and obesity and provides a mechanism for the association between fibrillin-1 and body fat. Since asprosin is produced from fibrillin-1 in white adipose tissue [13], it is consistent that individuals with more adipose would have higher FBN1 mRNA (as in Fig. 1 and Table 1). It may also explain why some Marfan patients (such as our case with an exon 25 mutation [8]) have reduced adipose regardless of dietary intake while others have normal or even excess adipose and consequent type II diabetes (for example some members of the family described in [67]), since the production of asprosin would depend on whether the furin site was able to be cleaved to produce the C-terminal fragment. For individuals with haploinsufficient mutations, those with deletions of the 3′ end of the gene, premature termination mutations or mutations that alter the structure to hide the site, the production of asprosin would be limited. In contrast, if a normal level of the fibrillin-1 protein is made (albeit dysfunctional) and the site is exposed and available, asprosin level would be regulated by external factors such as diet.

It remains to be determined whether the disappearance of fibrillin-1 mRNA and protein during synchronised adipogenesis *in vitro* is a cause or a consequence of differentiation. In a mouse model of Marfan syndrome, many of the phenotypic effects have been attributed to function of fibrillin-1 in regulating the bioavailability of transforming growth factor β (TGFβ) family members [68], [69], (including activins and bone morphogenic proteins) which inhibit adipogenesis [70], [71]. One role of fibrillin-1 may be to sequester these molecules during the initial stage of precursor commitment to differentiation. Interestingly, three chromosome 15 loci in the same general region as *FBN1* have been associated with body fat distribution [72], and one of these contains the gene encoding SMAD6, an inhibitor of TFGβ family member signalling [73] which has been associated with vascular disease [74]. Fibrillin-1 also has an important structural function in the extracellular matrix. Remodelling of the extracellular matrix from fibrillar to laminar type accompanies adipose differentiation [75] and is necessary to accommodate the altered shape and functions of the mature adipocytes. Proteins within the preadipocyte matrix such as fibronectin and presumably fibrillin-1 support an elongated fibroblast-like structure [76] which must be converted to the spherical shape of the mature adipocyte. Fibronectin forms the basis for fibrillin-1 assembly (reviewed in [77]) and its mRNA was down-regulated ahead of *FBN1* mRNA and protein. The reduction in fibrillin-1 protein may reflect the generalised down-regulation of connective tissue proteins during ECM remodelling. This remodelling is mediated by proteolytic degradation [78] which is consistent with the increased levels of protease mRNAs described here as differentiation proceeded.

Our results suggest that fibrillin-1 has a role in adipogenesis, and that it could mediate a genetic influence on body fat distribution [39] *via* a mechanism involving expansion of adipocytes, triggered by the newly discovered cleavage product asprosin [13]. Two lines of indirect evidence support this: there was a correlation between fibrillin-1 expression level and amount of adipose tissue in mouse and human, and *FBN1* mutations, particularly mutations in Exon 64 of the gene, impact on human fat deposition. The pattern of expression was similar to other ECM proteins including fibrillary collagens and fibronectin [26]. The relatively high level of mRNA seen in mature adipose tissues presumably reflects the constant turnover of ECM to maintain lipid storage capacity, and the production of the glucogenic cleavage product. This suggests that the lack of adipose tissue and consequent body image issues of MFS and lipodystrophy patients could be addressed by treatment with drugs that simulate the effect of the cleavage product. However this possibility must be supported by direct mechanistic experiments in fat cells or animal models. In addition it would be valuable to record measures of body fat status of patients with *FBN1* mutations, so that the mutations which predispose to lipodystrophy can be further characterised.

## 5 Conclusions

Down-regulation of *FBN1* expression is associated the transition from stem cell to preadipocyte to adipocyte, although a maintenance level of fibrillin-1 in adipose tissue is necessary and likely responsible for an endocrine response to low dietary gulcose. Targeting of *FBN1* or the fibrillin-1 protein may provide a therapeutic avenue for conditions where there is a deficiency of adipose tissue (such as Marfan syndrome and lipodystrophy) and for obesity and type II diabetes, responsible for a major health burden in today's world.

## Author contributions

MRD carried out the analysis of fibrillin-1 protein, validation of gene expression results and study of evolutionary conservation of the FBN1 gene, wrote the first draft of the manuscript and edited the final drafts. EA, ID and PA generated the adipose differentiation data for the FANTOM5 consortium and the human microarray data for obese and normal women and edited the manuscript. CRED and PDS provided access to human adipose derived mesenchymal stem cells and carried out the initial analysis of these cells and edited the manuscript. KMS initiated the project, performed the analyses of mouse and human gene expression data and wrote the manuscript.

## Conflicts of interest

The authors declare that they have no conflicts of interest with this work.

## Figures and Tables

**Table 1:** Correlation of *FBN1* expression with markers of adiposity in women.

*FBN1 vs*	Number of samples	Correlation coefficient (*r*)	Regression coefficient (*r*^*2*^)	Significance (*p*)
BMI	114	0.236	0.056	0.0114
Percent body fat	103	0.324	0.105	0.0008
Cell volume	114	0.284	0.081	0.0022
Fat cell number	114	0.008	6.37 × 10^− 5^	0.9328
